# Supplementary material for: Non-Contact Measurement of Thermal Diffusivity in Ion-Implanted Nuclear Materials
Source: Sci Rep. 2015 Nov 3;5:16042. doi: 10.1038/srep16042 (PMC4630606; doi:10.1038/srep16042)
Supplement: Supplementary Information [file srep16042-s1.pdf]

Non-Contact Measurement of Thermal Diffusivity in Ion-Implanted Nuclear Materials: Supplementary information

F. Hofmann[1]*, D.R. Mason[2], J.K. Eliason[3], A.A. Maznev[3], K.A. Nelson[3], S.L. Dudarev[2]

[1] Department of Engineering Science, University of Oxford, Parks Road, Oxford, OX1 3PJ, UK

[2] CCFE, Culham Science Centre, Abingdon, OX14 3DB, UK

[3] Department of Chemistry, Massachusetts Institute of Technology, 77 Massachusetts Avenue, Cambridge, MA 02139, USA

* *felix.hofmann@eng.ox.ac.uk* *+44 1865 283 446*

Supplementary Figures:

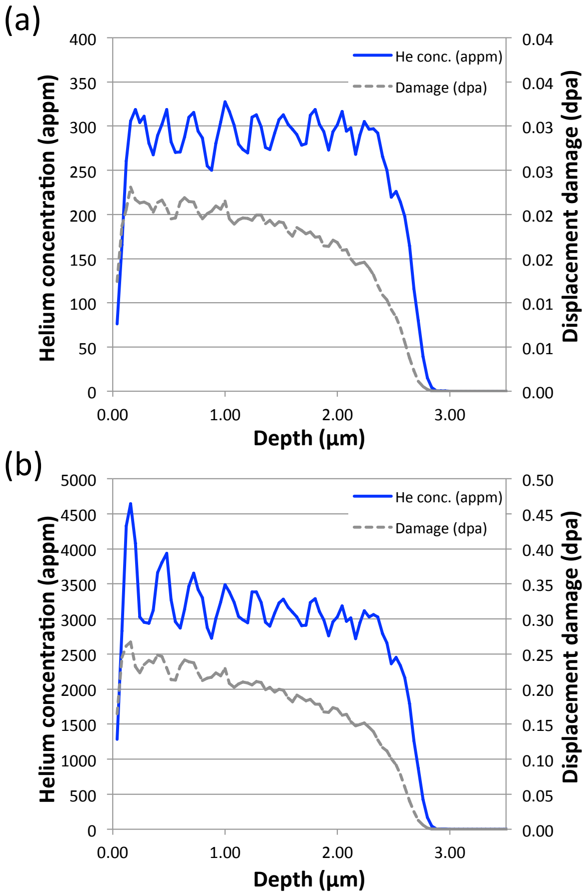

Supplementary Figure S1: Helium implantation and cascade damage profiles calculated using the Stopping Range of Ions in Matter code[1] for the low dose sample (a) and the high dose sample (b).

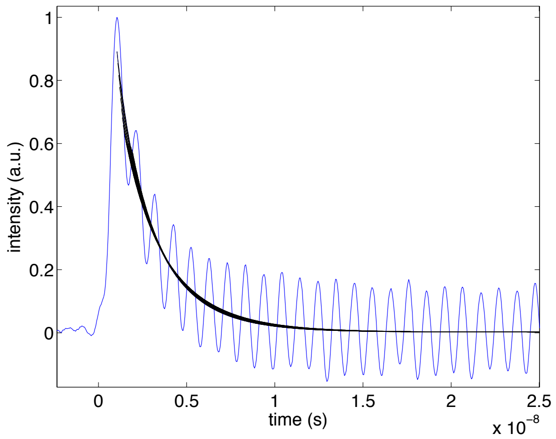

Supplementary Figure S2: Experimental data for pure tungsten at 296 K using λ = 2.74 µm (blue). Superimposed are experimental fits to the data using Eqn. S2 (black). Experimental fits were started at the maximum signal amplitude and up to 1 SAW oscillation period (~1 ns) later. In total 21 fits to experiments are shown.

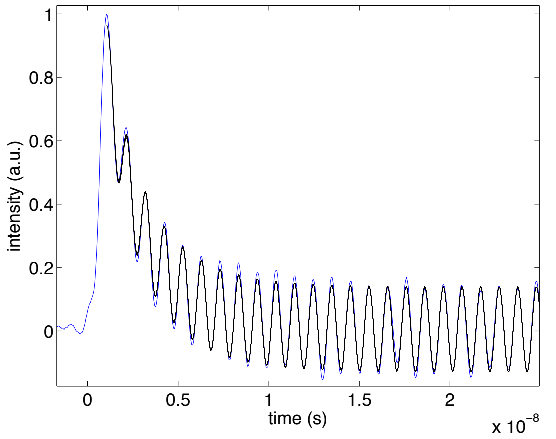

Supplementary Figure S3: Experimental data for pure tungsten at 296 K using λ = 2.74 µm (blue). Superimposed are experimental fits to the data using Eqn. S3 (black). Experimental fits were started at the maximum signal amplitude and up to 1 SAW oscillation period (~1 ns) later. In total 21 fits to experiments are shown.

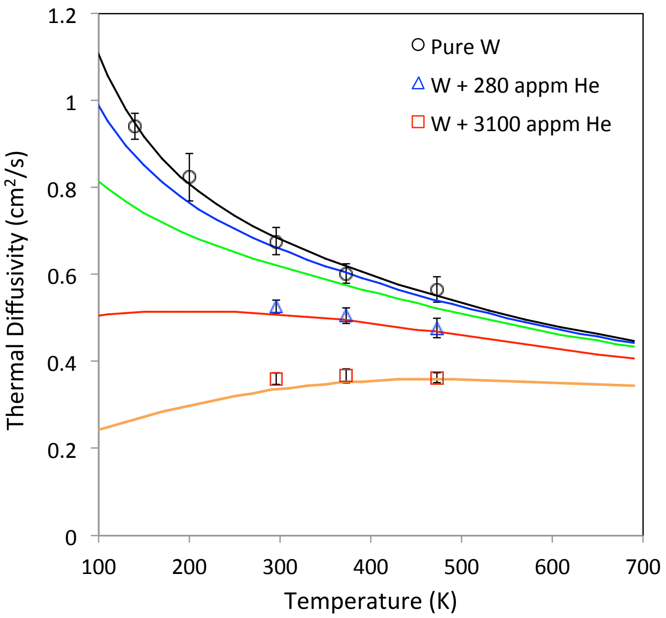

Supplementary Figure S4: Experimentally measured thermal diffusivity values for pure tungsten, tungsten implanted with 280 appm helium and tungsten implanted with 3100 appm helium (hollow symbols). Superimposed are predictions of thermal diffusivity calculated by treating vacancies and self interstitial atom defects as strong point scatterers. The lines represent: defect free material (black), 300 appm Frenkel pairs (blue), 900 appm Frenkel pairs (green), 3000 appm Frenkel pairs (red) and 9000 appm Frenkel pairs (orange).

Supplementary Methods:

1. Fitting of the experimental data:

The experimentally recorded signal in our measurements is dominated by the phase grating signal produced by displacement of the sample surface. The amplitude grating contribution, due to thermal reflectance, appears to be small, as discussed below. The decay of the surface displacement profile due to the thermal grating is given by[2]:

$$\frac{\partial u_z}{\partial x} \propto \text{erfc}\left(q\sqrt{\alpha t}\right), \qquad \text{(S1)}$$

where $q = 2\pi/\lambda$, λ is the thermal grating period, α is the thermal diffusivity and t is time. Fitting of the expression:

$$I = A * \text{erfc}\left(q\sqrt{\alpha t}\right) + G \qquad \text{(S2)}$$

to the experimental data could be used to quantify α, where A and G are parameters determined during the fit. However in this case the extracted value of α shows a significant dependence on the starting point of the fit. This is due to the large oscillations arising from the counter-propagating surface acoustic waves (SAWs), also generated by the pump beam, that are superimposed on the thermal decay signal. Fig. S2 shows an experimental data set for pure tungsten, measured at 296 K with λ = 2.74 µm. Superimposed are 21 fits to the data using Eq. S2, starting at different times, separated by 50 ps. The time delay between the earliest fit and the latest fit is thus 1 ns, corresponding to one SAW oscillation period. Clearly the lines of best fit do not fall onto the same curve. Indeed the normalised standard deviation, $\Delta\alpha/\alpha$, associated with the determined α values is ~6%.

The uncertainty in the value of α determined by fitting can be reduced significantly by including a term representing the sinusoidal SAW signal in the fitting procedure. Here the following expression was used:

$$I = A\text{erfc}\left(q\sqrt{\alpha t}\right) + C\sin(2\pi f t + E)\exp\left(-t/F\right) + G. \qquad \text{(S3)}$$

A, C, f, E, F, G and α are free parameters determined by fitting. Fig. S3 shows the fit of Eqn. S3 to the pure tungsten experimental data. Again 21 fits with starting times separated by 50 ps are shown. Clearly variation between the fits due to different starting positions is much smaller. This fitting procedure was used for all datasets

reported here. $\Delta\alpha/\alpha$, the fitting uncertainty, was consistently found to be on the order of 1%.

An important question concerns the appropriateness of neglecting thermo-reflectance in our data treatment. By varying the phase delay between the probe and reference beam, either the signal due to an amplitude grating (due to thermo-reflectance changes) or due to both amplitude and phase grating (due to surface displacement and thermo-reflectance changes respectively) can be probed[3]. We found the amplitude grating signal to be small compared to the phase grating signal. This indicates that the real part of thermo-reflectance is small. The combination of a small real part and large complex part of thermo-reflectance is unlikely. Hence the treatment of our data as arising only from a phase grating due to surface displacements induced by the temperature grating is appropriate. This is further confirmed by the fact that Eqn. S3 clearly constitutes a good fit to the experimental data, unlike other experimental data sets where the thermo-reflectance signal is important[4].

2. Evaluation of experimental uncertainties:

The uncertainty in the value of α due to fitting of the experimental data at a given measurement point is approximately 1%. A deviation of λ from the nominal value would introduce further errors. Here λ was calibrated by measuring the surface acoustic wave velocity for pure tungsten and adjusting λ to match the literature value for pure tungsten[5]. This calibration is aided by the fact that tungsten is almost perfectly elastically isotropic at room temperature[6-8]. There is also some point-to-point variation in the measured value of α for a given sample, typically on the order of 4%. The error bars shown in Fig. 2 and Fig. 3 of the main text were computed taking all of these error sources into account. Three measurements were recorded per temperature for pure tungsten at 140 K and 200 K. For all other datapoints statistics are based on 15 measurements per sample per temperature.

3. Kinetic theory model:

The principal carriers of heat in a reasonably pure metal at or above the Debye temperature (312 K in tungsten[9]) are electrons. Thermal diffusivity, α, can therefore be approximated as:

$$\alpha = \frac{\kappa_e}{C_{ph} + C_e}, \qquad \text{(S4)}$$

where C_{ph} is the Debye heat capacity and C_e the electronic heat capacity. κ_e is the electronic thermal conductivity. C_{ph} is given by:

$$C_{ph} = 9Nk\left(\frac{T}{T_D}\right)^3 \int_0^{\frac{T_D}{T}} \frac{x^4 e^x}{\left(e^x - 1\right)^2} dx\,, \tag{S5}$$

where k is the Boltzmann constant, T is temperature and T_D is the Debye temperature and N is the number of atoms per unit volume, i.e. $N = 1/\Omega_0$, where Ω_0 is the atomic volume (31.704 Å^3 for tungsten). The electronic heat capacity, C_e, is approximately given by:

$$C_e = c_e T\,, \tag{S6}$$

where c_e is a constant. The electronic thermal conductivity, κ_e, is:

$$\kappa_e = \frac{1}{3} C_e {v_F}^2 \tau_e. \tag{S7}$$

Fermi velocity, v_F, and electronic heat capacity, C_e, for metals can be measured or computed by Density Functional Theory[10]. In the dilute alloy limit, thermal diffusivity can be attributed to changes in the electron scattering time τ_e. From Matthiessen's rule, the total electron scattering rate is the sum of rates of scattering from impurities, phonons and other electrons, subject to the Ioffe-Regel limit that the electron mean-free-path cannot be much smaller than the separation between atoms[10,11]:

$$\tau_e = \sqrt{3/4}\, a_0/v_F + \left(\frac{1}{\tau_{imp}} + \frac{1}{\tau_{e-ph}} + \frac{1}{\tau_{e-e}}\right)^{-1}. \tag{S8}$$

Above the Debye temperature the electron-phonon scattering rate is proportional to the number of phonons, $1/\tau_{e-ph} \simeq \sigma_1 T$. The electron-electron scattering rate is proportional to the number of thermal electrons and holes, $1/\tau_{e-e} \simeq \sigma_2 T^2$. Scattering from impurities will be temperature independent, $1/\tau_{imp} \simeq \sigma_0$ [12]. Here σ_o, σ_1 and σ_2 are experimentally determined constants.

At alloying atoms or point defects in the matrix, impurity scattering has a new rate $\sigma_{0,m}$ so

$$\tau_{e,m} = \sqrt{3/4}\, a_0/v_F + \left(\sigma_{0,m} + \sigma_1 T + \sigma_2 T^2\right)^{-1}. \tag{S9}$$

The thermal conductivity for a matrix containing different types of defects or impurities, each with atomic fraction c_m and scattering time $\tau_{e,m}$, is then given by:

$$\kappa_e = \frac{1}{3} C_e v_F{}^2 \left(\sum_m \frac{c_m}{\tau_{e,m}} + (1 - \sum_m c_m) \frac{1}{\tau_e} \right)^{-1} \tag{S10}$$

Using v_F = 9.5 Å/fs, C_e/T = 5.5 x 10^{-10} eV/K^2/Å^3 and a_0 =3.165Å [10], a good fit to the measured diffusivity of pure tungsten is achieved with parameters (σ_0, σ_1, σ_2)=(3.09x10^{-3} fs^{-1}, 8.40x10^{-5} K^{-1}fs^{-1}, 2.11x10^{-7} K^{-2}fs^{-1}) (see Fig. 2 in the main text). The diffusivity data of W-Re alloys can be fitted with $\sigma_{0,Re}$ = 1.38±0.1 fs^{-1}. The change in electrical resistivity, ρ, due to a single rhenium atom in a tungsten matrix can be estimated using the Wiedemann-Franz law[12]:

$$\rho = \frac{L\,T}{\kappa_e}. \tag{S11}$$

At low temperature, using Lorenz number L = 2.44x10^{-8} W Ω K^{-2}, we find $\delta\rho_{Re}$ = 127±10 μΩcm/atomic fraction.

4. Modelling vacancies and self-interstitials as point scatterers:

Naively vacancy and self-interstitial atom defects could be modelled as strong point scatterers. Their measured electrical resistivities, $\delta\rho_v$ =700 μΩcm/at.fr. and $\delta\rho_i$ = 2000 μΩcm/at.fr. in tungsten respectively[13], can be converted to scattering rates, σ_v = 7.6 fs^{-1} and σ_i = 21.6 fs^{-1} respectively. These can then be substitute into the kinetic theory model described in supplementary section 3. Curves calculated using this approach, predicting the variation of thermal diffusivity with temperature, are shown in Fig. S4 for Frenkel pair concentrations of up to 9000 appm. Comparing these curves to the experimental data plotted in Fig. S4 suggests Helium : Frenkel pair ratios of 1:10 (sample implanted with 280 appm He) and 1:3 (sample implanted with 3100 appm). This is surprising, since previous experimental[5] and theoretical[14] studies indicate that generally only retention of a few Frenkel pairs per injected helium ion is expected. Closer inspection of equation S9 reveals that the high values of σ_v and σ_i yield an electron mean free path that is shorter than the interatomic distance, meaning that the scattering rate is actually dominated by the Ioffe-Regel limit. This means that simply treating vacancies and self-interstitial atom defects as point scatterers will substantially underestimate their effect on thermal diffusivity. Instead, for a more accurate estimate, the increased electron scattering rates at atomic sites in the vicinity of both defects must be accounted for as described in supplementary section 5.

5. Empirical atomistic model:

The calculated thermal diffusivity for He- implanted W shown in Fig. 3 was generated using an empirical atomistic model[10]. The procedure has two steps: Generating the configuration of defects, and then computing the thermal conductivity and heat capacity as a function of temperature.

We have chosen to represent the He-implanted material as an elastically relaxed random configuration of vacancy- and interstitial- point defects. The lattice defects introduced by the He ion bombardment will inevitably evolve with the thermal history of the sample, and it remains a serious theoretical challenge to unambiguously describe this process. Our simplification allows for a clear comparison between different implantation concentrations, without making assumptions about the true microstructure at the point of the experiment. As noted in the main text, He atoms within a vacancy do not contribute valence electrons, so are unlikely to act as scattering centres themselves.

The differences in elastic strain around a filled- and empty- vacancy will lead to small differences in the electron-phonon scattering rate, which can be neglected when compared to the Mott-Jones impurity scattering.

Defected configurations were generated by randomly removing atoms up to the concentrations specified from a 32x32x64 unit cell box of pure bcc tunsten crystal, then replacing them in crowdion interstitial positions, subject to the constraint that the interstitial atoms were not placed within half a lattice parameter of another point defect. The atoms were then relaxed using the Ackland-Thetford[15] EAM potential. The thermal diffusivity results shown are for a supercell with periodic boundary conditions volume-relaxed at zero temperature. Neglecting supercell relaxation or removing periodicity in the z-direction (resulting in a foil) changes the results by less than one percent.

The phonon heat capacity of the defected system is computed using the Debye approximation, assuming no change in the Debye temperature, as with the W-Re kinetic theory calculation. The electron heat capacity is computed atom-by-atom: for tungsten below the melting temperature we can use the Sommerfeld expansion,

$$C_{e,i} = \frac{1}{3}\pi^2 k_B{}^2 T_e D_i \,, \tag{S12}$$

where D_i is the local density of states of atom i, a by-product of the second moment approximation used in the EAM potential[10].

The inverse thermal conductivity is computed by summing the electron-electron, electron-phonon, and impurity scattering rates on each atom. The electron-electron scattering rate is fitted to the experimental curve for pure tungsten[9]. The electron-phonon scattering rate is computed by matching an expression for electron-phonon damping using the empirical potential to the experimental electron-phonon coupling factor[16]. The Mott-Jones impurity scattering model is a simple empirical fit: the enhanced scattering rate due to an atom in a defected configuration is taken to be proportional to its excess potential energy. This rate can then be fitted to reproduce the experimental electrical resistivity per Frenkel pair[13,17] using the Wiedemann-Franz law. Detailed expressions and fitting procedures for these three contributions are provided by Mason[10].

Supplementary References:

1 Ziegler, J. F., Ziegler, M. D. & Biersack, J. P. SRIM - The stopping and range of ions in matter (2010). *Nuclear Instruments and Methods in Physics Research, Section B: Beam Interactions with Materials and Atoms* **268**, 1818-1823; DOI:10.1016/j.nimb.2010.02.091 (2010).

2 Käding, O. W., Skurk, H., Maznev, A. A. & Matthias, E. Transient thermal gratings at surfaces for thermal characterization of bulk materials and thin films. *Appl. Phys. A* **61**, 253-261; DOI:10.1007/BF01538190 (1995).

3 Johnson, J. A. *Optical Characterization of Complex Mechanical and Thermal Transport Properties* PhD thesis, Massachusetts Institute of Technology, (2011).

4 Johnson, J. A. *et al.* Phase-controlled, heterodyne laser-induced transient grating measurements of thermal transport properties in opaque material. *Journal of Applied Physics* **111**, 023503; DOI:10.1063/1.3675467 (2012).

5 Hofmann, F. *et al.* Lattice swelling and modulus change in a helium-implanted tungsten alloy: X-ray micro-diffraction, surface acoustic wave measurements, and multiscale modelling. *Acta Materialia* **89**, 352–363; DOI:10.1016/j.actamat.2015.01.055 (2015).

6 Featherston, F. H. & Neighbours, J. R. Elastic Constants of Tantalum, Tungsten, and Molybdenum. *Physical Review* **130**, 1324-1333; DOI:10.1103/PhysRev.130.1324 (1963).

7 Lowrie, R. & Gonas, A. M. Single-Crystal Elastic Properties of Tungsten from 24° to 1800°C. *Journal of Applied Physics* **38**, 4505-4509; DOI:10.1063/1.1709158 (1967).

8 Bolef, D. I. & De Klerk, J. Elastic Constants of Single-Crystal Mo and W between 77° and 500°K. *Journal of Applied Physics* **33**, 2311-2314; DOI:10.1063/1.1728952 (1962).

9 Ho, C. Y., Powell, R. W. & Liley, P. E. Thermal Conductivity of the Elements. *Journal of Physical and Chemical Reference Data* **1**, 279-421; DOI:10.1063/1.3253100 (1972).

10 Mason, D. R. Incorporating non-adiabatic effects in Embedded Atom potentials for radiation damage cascade simulations. *Journal of Physics: Condensed Matter* **27**; DOI:10.1088/0953-8984/27/14/145401 (2015).

11 Wiesmann, H. *et al.* Simple Model for Characterizing the Electrical Resistivity in A-15 Superconductors. *Physical Review Letters* **38**, 782-785; DOI:10.1103/PhysRevLett.38.782 (1977).
12 Ziman, J. M. *Electrons and Phonons: The Theory of Transport Phenomena in Solids*. (Oxford University Press, 2001).
13 Ullmaier, H. *Atomic Defects in Metals*. Vol. 25 (Springer-Verlag, 1991).
14 Becquart, C. S. & Domain, C. An object Kinetic Monte Carlo Simulation of the dynamics of helium and point defects in tungsten. *Journal of Nuclear Materials* **385**, 223-227; DOI:10.1016/j.jnucmat.2008.11.027 (2009).
15 Ackland, G. J. & Thetford, R. An improved N-body semi-empirical model for body-centred cubic transition metals. *Philosophical Magazine A* **56**, 15-30; DOI:10.1080/01418618708204464 (1987).
16 Brorson, S. D. *et al.* Femtosecond room-temperature measurement of the electron-phonon coupling constant γ in metallic superconductors. *Physical Review Letters* **64**, 2172-2175; DOI:10.1103/PhysRevLett.64.2172 (1990).
17 Broeders, C. H. M. & Konobeyev, A. Y. Defect production efficiency in metals under neutron irradiation. *Journal of Nuclear Materials* **328**, 197-214; DOI:10.1016/j.jnucmat.2004.05.002 (2004).
